# What Makes *Cells* Different from Other Open Access Journals

**DOI:** 10.3390/cells6040041

**Published:** 2017-11-01

**Authors:** Alexander E. Kalyuzhny

**Affiliations:** Department of Neuroscience, UMN Twin Cities, 6-145 Jackson Hall, 321 Church St SE, Minneapolis, MN 55455, USA; kalyu001@umn.edu

In 2011, I was invited to serve as the Editor-In-Chief for *Cells*, which back then was a “new kid on the block” among open access (OA) journals. There are several reasons why I decided to accept the invitation, the most important one being the passion of MDPI and *Cells’* administrative team to create a first-class international forum for researchers studying the mechanisms underlying the structure and function of a cell. Having first-hand access to submitted manuscripts, I am privileged to see the latest scientific trends and discoveries. On the other hand, serving as the EIC is quite challenging and requires difficult decisions to be made. How should we proceed with a paper that has received mixed reviews ranging from “accept after minor revision” to “reject”? Is it worth adding a new Special Issue? Should the paper be recommended for peer review or not? Fortunately, thanks to our dedicated administrative staff, we manage to solve these issues in a timely and professional manner, which is a great example of team work.

As a scientist, I respect *Cells* for being so meticulous and thorough in choosing both editorial board members and reviewers who have made *Cells* a success story: our journal was accepted into the Science Citation Index Expanded (SCIE), the bibliographic database service BIOSIS, and the database Biological Abstracts. *Cells* is eligible for inclusion in next year’s Journal Citation Reports (JCR), the annual publication by Clarivate Analytics.

Despite *Cells’* success and steady rise in the ranks of OA journals, there is a lot of work that lies ahead. We want to improve our visibility and attractiveness to researchers worldwide, thus making our journal the science forum of choice, with expert reviewers and an expedited publication process. Globally, our principal focus will remain the same: cell structure and function with an emphasis on growth, differentiation, and aging. In particular, we will still be focusing on cell adhesion and motility, regulation of intracellular signaling, and physiology of cancer and stem cells. We are open to both original research papers and reviews. However, due to a constantly changing scientific landscape with rapidly emerging novel areas of research, we are placing a strong emphasis on Special Issues that allow us to present specific research in a highly condensed form. In the past, we have successfully introduced a variety of Special Issues that became very popular with our audience, serving as mini-forums in specific areas of cell science. We encourage researchers to suggest topics for Special Issues and to volunteer to serve as either guest editors or reviewers.

Regarding our short-term goals, it appears that there is a growing interest in identifying the biomarker signatures of cell subtypes, which for the most part remain unknown. There are ambitious global projects underway aimed at identifying the molecular characteristics of cells by employing both transcriptomics and proteomics techniques. These studies are going to have a dramatic impact because they will pave the way to unraveling the links among a variety of diseases and pathologies. I have every reason to believe that *Cells* is the perfect place for reporting novel findings in this area. Our long-term goals will remain unchanged: we are committed to making *Cells* a scientific forum where highly regarded researchers feel at home and can be proud of publishing their papers.

As for me personally, I will continue to do my humble EIC job and, to the best of my capabilities, will help with advancing cell science and attracting talented young investigators to this fascinating field of biology.

